# Development of the Health Incentive Program Questionnaire (HIP-Q) in a cardiac rehabilitation population

**DOI:** 10.1007/s13142-015-0330-3

**Published:** 2015-06-26

**Authors:** Marc S. Mitchell, Jack M. Goodman, David A. Alter, Paul I. Oh, Guy E. J. Faulkner

**Affiliations:** Faculty of Kinesiology and Physical Education, University of Toronto, 55 Harbord St., Toronto, ON M5S 2W6 Canada; Institute for Clinical Evaluative Sciences, Toronto, ON Canada; Toronto Rehabilitation Institute, University Health Network, Toronto, ON Canada

**Keywords:** Exercise, Incentives, Cardiac rehabilitation, Prevention, Motivation

## Abstract

The purpose of this study was to develop a questionnaire to facilitate the design of acceptable financial health incentive programs. A multiphase psychometric questionnaire development method was used. Theoretical and literature reviews and three focus groups generated a pool of content areas and items. New items were developed to ensure adequate content coverage. Field testing was conducted with a convenience sample of cardiac rehabilitation (CR) patients (*n* = 59) to establish face and construct validity (*p* = 0.021) and reliability (intraclass coefficients = 0.42–0.87). The final questionnaire is comprised of 23 items. This questionnaire builds on previous attempts to explore acceptability by sampling a wider range of instrumental and affective attitudes and by measuring the effect of program features on the likelihood of incentive program participation. Future research is now needed to examine whether tailoring incentives to preferences assessed by the questionnaire improves uptake and effectiveness.

## INTRODUCTION

The societal costs of chronic disease are enormous. Employers bear their share of this burden as they pay more for unhealthy employees in health costs, disability, and absenteeism expenses. In 2013, for example, US employers paid $9157 (US) per active employee in health costs—up from $7486 in 2009 [1]. This number is expected to increase by 4.4 % (twice the rate of inflation) in 2014. In response, many employers in the USA (and elsewhere) have added wellness programs to their package of benefits. Low levels of employee engagement have unfortunately been a hallmark of these programs. Web-based wellness programs are particularly susceptible to attrition [2]. To boost engagement, two thirds of large US employers now offer financial incentives for wellness program participation [1]. Companies are forging ahead with less than optimal incentive schemes, however, limiting returns-on-investment. For example, by offering incentives in the delayed, less salient form of health insurance premium reimbursements (61 % of US employers do so) [1] for the attainment of hard-to-achieve biometric standards (58 % of US employers do so) [1], companies risk squandering scarce resource on weak behavioral stimuli [3, 4].

To optimize incentive program design, the range of incentive program features should be considered in the design process (see Table 1 for a list of incentive design features and attributes). To date, not enough attention has been paid to these features even though they appear to moderate effectiveness [5]. Even subtle variations in incentive design, for example, can have a profound impact on target group “acceptability” [6, 7], a critical precondition to successful incentive program implementation [4]. Since incentives for health remain a contentious topic (about half of survey respondents think they are unfair, coercive, a breach of privacy, or a waste of limited resource) [8–14], a tool is needed to assess target group acceptability in advance of implementation. This tool could be used to identify acceptability moderators and preferred incentive features. Learning more about preferred incentive program features, and how these vary for individuals and groups with shared characteristics (e.g., older employees, lower income earners), should inform the design of more refined, effective, cost-effective, and marketable (e.g., “custom incentives”) incentive programs.

**Table 1:** Financial health incentive design features and the range of attributes for each (examples in parentheses)

Features	Attributes
1. Form	(a) Cash ($10 cash, cheque)
	(b) Voucher (iTunes, grocery, transit, Amazon)
	(c) Specific good/service (gym shoes, dietician consultation)
	(d) *Reimbursement* (existing expense reimbursed, like gym membership fee or health insurance premium)
	(e) *Donation* (value of incentive earned donated to charity of choice)
2. Magnitude	Continuous variable (often expressed as dollars (US) per week or month)^a^
3. Target	(a) *Self-regulatory behavior* (self-monitoring, scheduling, seeking social support)
	(b) Behavior (exercise, medication adherence)
	(C) Outcome (BMI < 25 kg/m^2^, BP < 140/90)
4. Timing of assessment	(a) Completion of incentive program (6 months)
	(b) Set intervals (daily/weekly assessments)
	(c) Random intervals (10 assessments over 6 months)
	(d) Dependent intervals (varying intervals based on previous performance)
5. *Type of assessment*	(a) *Self-report* (exercise diary submission)
	(b) *Objective, direct assessment* (face-to-face)
	(c) *Objective, indirect assessment* (pedometer, photo of weight on scale)
6. Reward immediacy^b^	Continuous variable (often expressed as days or weeks between assessment and reward)
7. Certainty	(a) Certain ($50 for meeting A1C target)
	(b) Certain chance (1 in 4 chance of $25)
	(c) Uncertain chance (1 in 100 chance of $500)
	(d) Mix ($50 and a 1 in 100 chance of $500)
8. Schedule	(a) Uniform ($50 lump sum for meeting goal)
	(b) Indexed ($1 for each gym visit)
	(c) Escalating ($1 for first 10 gym visits, $2 for next 10, etc.)
	(d) Random ($1 to $50 for gym visits)
9. Dispensing type	(a) Resetting (discreet reward at time of each achievement)
	(b) Aggregative (“passbook saving”—information on running tally given)
	(c) *Mix* (accumulated incentives lost if discreet goal not met, “Go back to zero if missed gym visit.”)
10. Participant investment	(a) Opportunity cost only (time)
	(b) Deposit contract (own money lost if fail to achieve goal)
	(c) Matching (“double or nothing”) ($50 of own money lost if fail, $50 extra gained if successful)
11. Information disclosure	(a) Factual (information given about meeting or failing to meet goal)
	(b) Counterfactual (information given about reward lost by failing to meet goal, i.e., regret)
12. *Duration*	Continuous variable (often expressed in weeks or months incentive available; maybe indefinitely)
13. *Source*	(a) *Self or significant others* (spouse, friend)
	(b) *Group members* (incentive plan members)
	(c) *Government*
	(d) *Employer*
	(e) *Insurance company*
	(f) *Other (noninsurance) companies*
14. Recipient	(a) Individual (cash for weight lost)
	(b) Group (reward for >50 % group attendance)
	(c) Significant other(s) (spouse, parent)
	(d) *Charitable organization*

This table represents a combination of works published by Klein and Karlawish [24], Adams et al. [25], and Mitchell and Faulkner [2]. Italicized items have not been previously published

^a^Magnitude is ideally considered in relation to individual/group socioeconomic circumstance

^b^Consider when (1) behavior/outcome is assessed, (2) when it is rewarded, and (3) the time between assessment and reward

Therefore, the purpose of this study was to develop a new survey, called the Health Incentive Program Questionnaire (HIP-Q), to measure target group acceptability and identify preferred incentive program structures. Despite conducting this study with a convenience sample of cardiac rehabilitation (CR) patients, lessons learned may be applicable to employee-directed incentives as well. Incentives that are more readily accepted by target groups may lead to greater program uptake, increase the potential for sustained behavior change, and ultimately yield cost-effective outcomes.

## METHODS

A multiphase psychometric questionnaire development method was used to develop the HIP-Q [15]. The HIP-Q is designed specifically to optimize incentives *for exercise*, since the authors of this study planned to deploy the questionnaire for the first time in a CR context, where exercise is the cornerstone therapy. Of all the chronic disease risk factors, physical inactivity is arguably the most important [16] and has been recently shown to increase health care costs in US employees with metabolic syndrome by about 30 % [17]. Incentives can target any number of health behaviors though (e.g., medication adherence, weight loss) and the HIP-Q was designed to easily incorporate them (e.g., by replacing the word “exercise” with “take my medications” or “lose weight” in HIP-Q item #1 stem: “For me, getting paid cash or healthy vouchers to exercise/take my medications/lose weight *would be…*” A convenience sample of English-speaking CR participants was recruited from the Toronto Rehabilitation Institute’s (Toronto Rehab) to participate in the development of the HIP-Q. Participants were recruited between February and October 2013 by a researcher at the beginning of their weekly CR sessions. The sample broadly reflected the sociodemographic profile (e.g., gender, marital status, income) of cardiac patients in Ontario (see Table 2 for population and sample characteristics). Drawing from the Toronto Rehab CR population was deemed appropriate since it is suggested that high-risk, high-cost groups, such as older adults living with cardiovascular disease, should be the initial targets of incentive interventions [18, 19]. The research ethics boards of the University Health Network and the University of Toronto approved this study. The development of the questionnaire consisted of five phases.

**Table 2:** Population and sample sociodemographic characteristics

	Population^a^, *n* = 1807	Sample, *n* = 59
Age (years, mean ± SD)	65.4 ± 10.4	66.0 ± 10.9
Female	450 (25)	13 (22)
Caucasian	1446 (83)	47 (80)
Married	1392 (78)	37 (63)
Retired	905 (52)	36 (61)
Postsecondary education	1312 (75)	43 (73)
Household income (Canadian dollars)		
<$35,000	N/A	12 (20)
<$65,000	N/A	27 (46)
>$65,000	730 (50)^b^	32 (54)
>$95,000	N/A	22 (37)

Numbers in parentheses represent the percent of participants within the condition (column) possessing the given attribute

^a^Sociodemographic characteristics of cardiac inpatients from 11 Ontario hospitals enrolled in the Cardiac Rehabilitation Care Continuity through Automatic Referral Evaluation study (1807 out of 2635 recruited patients were enrolled) [36]

^b^Annual family income >$50,000 (Canadian)

### Phase 1: identifying content areas and items

#### Step 1—literature review

A review of relevant behavior change theories served a “heuristic purpose” [15] suggesting content areas that the authors could use to begin to shape the HIP-Q as well as to identify and/or phrase items. A systematic review of the literature examining incentives for exercise adherence in adults [20] and an overview of related papers and reviews also added to the inventory of content areas and items.

#### Step 2—focus groups

Three focus groups of five to six CR participants were conducted to explore opinions of incentives, and elucidate content areas and items that may not have emerged from the theoretic/literature reviews. The focus group methodologies have been previously reported [21].

### Phase 2: new item generation

#### Step 3—drafting new items

New items were developed to ensure adequate coverage in the HIP-Q. The 14 features of incentive programs in Table 1 were used to guide this step, ensuring all features (and attributes) were considered in the incentive design process.

### Phase 3: content validity

#### Step 4—expert consultation

Once new items were written, a draft of the HIP-Q and an accompanying review guide were sent to five international experts with experience conducting incentive research, writing about incentives, implementing incentives, developing surveys, and/or working with CR patients. Four out of five experts held Ph.D. degrees in health behavior change or related fields. In the review, guide experts were asked about the appropriateness and clarity of items using a 4-point Likert scale. Items with mean scores below 3 were discarded or reconsidered. Experts were also asked if the HIP-Q sampled all relevant content given its stated purpose, and to recommend additional content areas/items, if needed. Lastly, experts were asked to recommend different approaches to scaling, and to reword items, as required.

### Phase 4: face validity

#### Step 5—pretesting

The HIP-Q was pretested in one-on-one interviews to ensure that it was comprehensible for the target population before pilot testing it with a larger group. To explore whether the questions were clear, individuals were asked to “think aloud” through their responses to identify problem items. Problem items were rewritten or eliminated. Frequency of endorsement was tested and discarding item alternatives was considered if endorsed by very few or very many (endorsement rate of 0.20 and 0.80, respectively). Pretesting continued until no new concerns arose. Time for HIP-Q completion was recorded.

### Phase 5: construct validity and reliability

#### Step 6—pilot testing

The final draft of the HIP-Q (23 total items) was piloted through paper-and-pencil self-administration in a convenience sample of 59 CR patients to test construct validity and reliability. In line with self-determination theory, the authors hypothesized that individuals scoring lower on the Relative Autonomy Index (RAI; summary measure of intrinsic motivation to exercise calculated using the Behavioral Regulation to Exercise Questionnaire (BREQ-3)) [22] would favor incentive program participation, as indicated by a “likely” or “very likely” response to HIP-Q item #2: “In general, how likely would you be to participate in an incentive program that paid you $40 a month for exercising 15 min a day, 3 days a week?”

### Statistical analyses

The magnitude and statistical significance of the relationship between the RAI and the “likelihood of participation” response was evaluated using Pearson’s correlation coefficient. To test reliability, a 7-day test-retest was conducted and intraclass coefficients (ICC) were computed. Identifying the number of patients leaving more than 10 % of items unanswered also tested completeness of item responses, or who incorrectly answered items. All data analyses were conducted using the Statistical Package for the Social Sciences 22.0 (SPSS).

## RESULTS

### Phase 1: identifying content areas and items

#### Step 1—literature review

The theoretical review conducted during phase 1 highlighted the value of grounding incentives in health behavior change theory. A full outline of the theoretical considerations informing the development of the HIP-Q is reported elsewhere [23]. In keeping with self-determination theory in particular, for incentives to drive sustained health behavior change, the authors suggest incentives be designed in a way that fulfills the basic psychological needs of competence (experiencing mastery), autonomy (a sense of ownership over behavior), and/or social relatedness (feeling socially connected to others). The HIP-Q therefore included items that aimed to identify health behaviors or outcomes that prospective participants could realistically achieve (to increase confidence). The HIP-Q’s purpose was to aid in the delivery of custom incentives to increase feelings of ownership and autonomy. Last, incentives related to social outcomes (e.g., charitable donations) or that promote social interaction (e.g., incentives for group success) were included in the HIP-Q as plausible program options. The three psychological needs described in self-determination theory were carefully considered, therefore, in the development of the HIP-Q.

The literature review undertaken by the authors [20] uncovered two papers that outlined and defined the set of incentive design features (and their associated attributes) [24, 25]. So not to neglect important features in the design process, the HIP-Q was formatted according to these published features (there are 11 in total), as well as three additional features emerging from the authors’ review [20] (i.e., type of assessment, duration of incentive program, source of incentive; see Table 1), with the aim of using the data to customize incentive programs. According to a 2013 Consensus Statement, *Guidance for a Reasonably Designed, Employer-Sponsored Wellness Program Using Outcomes Based Incentives*, to build employee acceptance all “reasonably designed” incentive programs should consider the full range of incentive approaches when looking to increase wellness program uptake and engagement [26]. In addition, a questionnaire developed and used by Long et al. to examine opinions of incentives was discovered during the literature review phase of this study [27]. This questionnaire was not validated but provided a foundation from which to build an updated, more comprehensive, and psychometrically sound incentive-focused questionnaire. The questions developed by Long et al. were used as the initial basis for the HIP-Q items.

#### Step 2—focus groups

The focus group results have been previously reported [21]. Briefly, a thematic analysis of the focus group data revealed that participants’ ethical concerns with incentives were prominent, but were mitigated in considering a range of program features, including source (e.g., government vs. private company) and type (e.g., cash vs. voucher) of incentive, as well as incentive target (e.g., behavior vs. outcome) (see Appendix 1 for an overview of focus group themes and acceptability moderators). Identifying the features most likely to elicit strong (negative) reactions in this sample focused the authors’ attention on key content areas (i.e., design features), ensuring that these areas/features were adequately addressed in the questionnaire.

### Phase 2: new item generation

#### Step 3—drafting new items

Since ethical concerns were prominent in the focus groups (consistent with the literature) [6–13], the Long et al. questionnaire was expanded using Spector’s and Ajzen’s lists of categories to include seven total pairs of instrumental (e.g., Necessary/Unnecessary) and affective (e.g., Fun/Not Fun) attitudes. A paired comparison technique was used here, where respondents were asked to indicate which attitudinal opposite they agreed with most on a 7-point Likert scale (uneven to offer a “neutral position,” and with most points labeled to ease cognitive requirements) [15].

A paired comparison technique using 7-point Likert scales was also used to identify features that may increase the “likelihood” of incentive program participation as well as to identify preferred incentive design features. The “likelihood of participation” and incentive design preference items were deemed to be more directly relevant for employers and others interested in investing in incentives for health than broader attitudinal items. Notably, it was not suitable for every incentive design feature from Table 1 to be represented in the HIP-Q. In particular, new items exploring features #4, #8, and #9 (timing of assessment, schedule, and dispensing type) were not drafted given the overlap with feature #6 (reward immediacy). To further limit redundancy, feature #5 (type of assessment, e.g., self-report) was not explicitly represented in the HIP-Q either given similarities with feature #3 (incentive target, e.g., self-monitoring).

One categorical item was drafted to identify specific voucher preferences, since vouchers may be perceived as more acceptable and meaningful than cash alone [21]. In total, 28 new items were drafted (replacing the Long et al. items) to accommodate a more comprehensive assessment of attitudes around incentives and to determine whether acceptability varies with design features/attributes. Several steps were taken to ensure that the newly devised items were psychometrically sound including using words that do not require greater than a 6th grade reading level.

### Phase 3: content validity

#### Step 4—expert consultation

Mean appropriateness and clarity scores, as given by content experts, ranged from 3.2 to 4.0, and thus, no items were discarded due to low scores. Seven items were edited, as per reviewer suggestions, to increase clarity. To ease cognitive requirements, the number of response alternatives for the paired comparisons was reduced to five (from seven). The instructions and stems in this section were also edited for clarity. The depth to which certain items explored the role of design feature attributes in moderating acceptability (e.g., certain vs. uncertain rewards) was deemed to be unnecessarily complex, and potentially confusing, by three experts, and so these items were rewritten. Once recommendations from experts were incorporated, a “readability score” (7.6 Fleisch-Kincaid Grade Level) was generated using Microsoft Word. This score was interpreted with caution given some of the limitations outlined by Streiner and Norman [15].

### Phase 4: face validity

#### Step 5—pretesting

**Fig 1:**
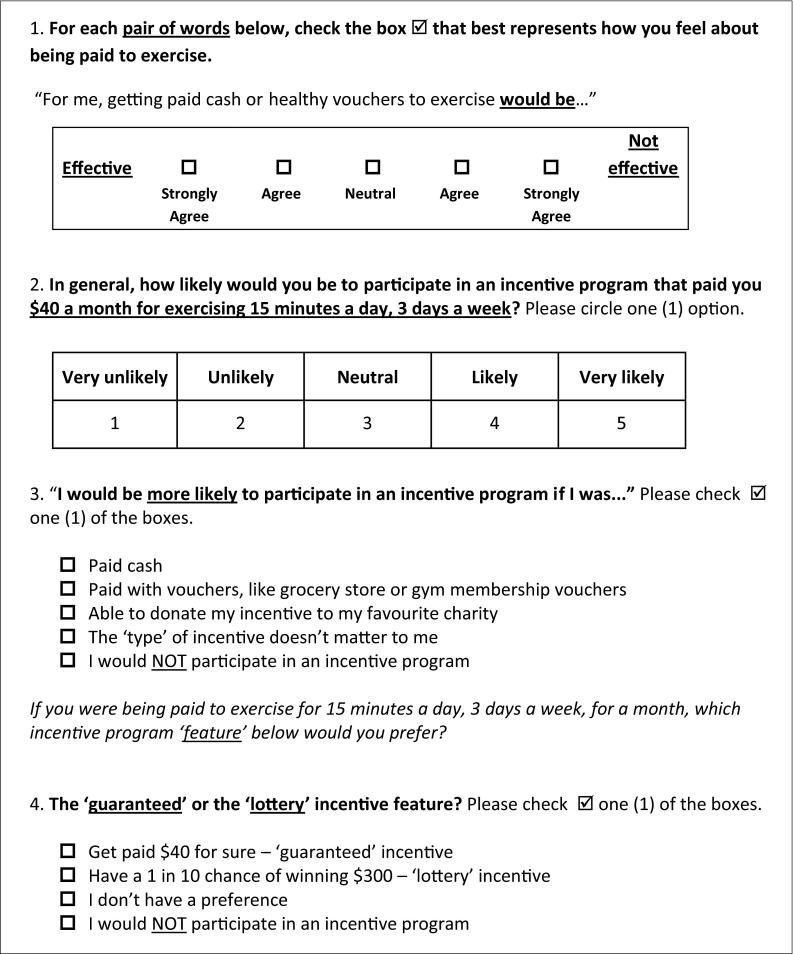
Sample Health Incentive Program Questionnaire (HIP-Q) items

Eight participants completed the HIP-Q and participated in one-on-one interviews. No new concerns arose during the final three interviews and so sampling ceased at this point. Missing values on one or more items occurred in three participants (37.5 %). Paired comparison items (for instrumental and affective attitudes) were reformatted to include *labeled check boxes* (see Fig. 1, or the full HIP-Q in Appendix 2), rather than numbers (1–5) to be circled, as the inherent values of numbers confused some of the participants (e.g., “So ‘1’ is the highest?”).

**Fig 2:**
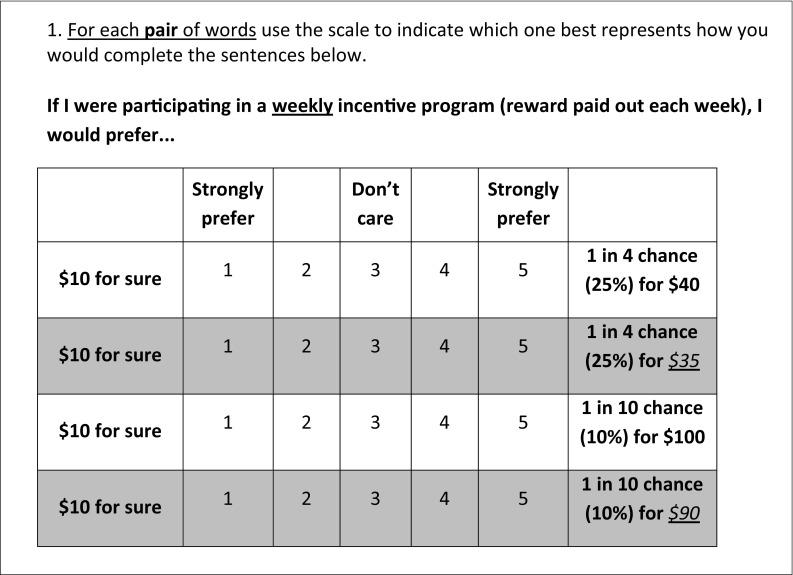
Item from the “pretesting” draft of the Health Incentive Program Questionnaire using paired comparisons to examine the impact of subtle feature attribute variations on incentive program preferences

Using paired comparisons to examine the impact of *subtle* feature attribute variations on opinions confused some participants (see Fig. 2). For this reason, questions about feature attribute preferences were reformatted to simpler categorical judgments (see Fig. 1, item 4), with fewer “variations” presented, bringing the total number of HIP-Q items to 23, from 28. Endorsement rates of item alternatives did not fall outside a priori parameters and so no item alternatives were eliminated. The average time to completion was 13 min 21 s.

### Phase 5: construct validity and reliability

#### Step 6—pilot testing

The HIP-Q was pilot tested with CR patients through self-administration to test construct validity (*n* = 59) and reliability (*n* = 32). Seventy-one percent (17/24) of the respondents with RAIs below the group mean (i.e., more externally controlled—“I exercise because my doctor told me to.”) indicated that they would be likely/very likely to participate in an incentive program compared to 51 % (18/35) of those above the mean (e.g., “I exercise because I enjoy it.”). As well, RAI and likelihood of participation were correlated (*p* = 0.021) supporting the authors’ a priori hypothesis that less self-determined respondents would self-report being more likely to participate in an incentive intervention. An examination of BREQ-3 subscales yielded similar results, with 71 % of more “externally regulated” respondents (15/21, vs. 52 % of those less “externally regulated”) indicating they would be likely/very likely to participate in an incentive program. Ten (16.9 %) respondents either did not answer, or incorrectly answered, 10 % or more of the items. For instrumental and affective attitude items, the ICCs were 0.76 and 0.60, respectively. For categorical items, the ICCs ranged from 0.42 to 0.87 (see Fig. 1 for a sample of HIP-Q items and Appendix 2 for the full questionnaire).

## DISCUSSION

The aim of this study was to develop a valid and reliable questionnaire for the purpose of customizing health incentives. This is the latest attempt to develop a novel incentive design tool, the first study to consider the broad range of incentive design features in the development of such a tool, and the first to psychometrically evaluate a health incentives program questionnaire. Although this study was conducted in a CR context, there is no obvious reason that the HIP-Q cannot be applied in other contexts and for other health behaviors given its focus on core design features of incentives. Preferences may certainly vary across populations and contexts, and further validation work will be needed to demonstrate this.

### Psychometric properties

The HIP-Q demonstrated content, face, and construct validity. Informed by the extant literature and expert review, the HIP-Q adequately covers the relevant information. As well, items were interpretable by the target population during pretesting, and pilot testing demonstrated that the HIP-Q (item #2) is significantly related to RAI (calculated using the BREQ-3) [22], consistent with self-determination theory, increasing confidence in responses. HIP-Q test-retest reliability was partly supported as well, with 12 out of 23 items demonstrating “Good” reliability (ICC ≥ 0.7). Since the purpose of the HIP-Q was to assess as many design features as possible, items with less than satisfactory reliability (*n* = 11; ICC < 0.7) were not discarded. Those interested in implementing incentives should interpret item responses with caution until further validation is conducted. Notably, affective attitude items (e.g., Good vs. Bad) yielded different responses over time (ICC = 0.60). Affective attitudes around incentives may be nebulous, changing over time, perhaps with the presentation of new information, or in different settings, or with more time for personal reflection on a contentious topic. Before drawing firm conclusions regarding the reliability of these items, further study is warranted with a larger sample.

### Application

The HIP-Q is a comprehensive incentive design tool that has several potential applications. Since low program uptake is a barrier to successful implementation, the HIP-Q may be used to identify the overall acceptability of interventions. Although effectiveness was not tested in this article, the authors presume that higher acceptance of incentive designs may lead to greater effectiveness, as has been suggested [4]. Not only may the HIP-Q be used to identify perceived levels of effectiveness and acceptability (instrumental and affective attitudes), but it may also be used to establish how likely individuals would be to sign-up for an incentive program. The HIP-Q allows for the identification of feature attributes that may boost likelihood of participation as well, providing incentive program sponsors with information to customize incentive packages so they are more readily accepted by target groups. Building a repository of incentive program design preferences over time and linking these to sociodemographic, health status, and health behavior characteristics may help segment incentive interventions in the future.

The HIP-Q may also help shed light on the question of “incentive direction” (i.e., Should companies implement financial health incentives, or penalties?). For instance, HIP-Q items #6 and #7 ask respondents how likely they would be to “wager” their own money in an incentive program (called a “deposit contract”). The answers to these questions may give companies a sense of how willing employees would be to pay an enrollment fee, with the chance to earn their money back in the program. This incentive structure is becoming increasingly common [1] and is the one championed in the Patient Protection and Affordable Care Act—where employees can earn back up to 50 % of their health insurance premium (their “deposit,” so to speak) [28]. Regarding the implementation of a financial penalty over and above the cost of insurance, our position is that penalties are more likely to generate resistance [29], limit enrolment [29, 30], discriminate disadvantaged groups [31], and undermine intrinsic motivation [23]—damaging the potential for sustained health behavior change [32].

Regarding tailored incentive programs, a growing body of research is examining how individual characteristics (e.g., age, income, confidence to exercise, weight status, consumer habits/preferences) moderate incentive effectiveness, and how these characteristics interact with incentive design features/attributes to produce health behavior change. For instance, John et al. determined in a sample of low and high income adults that the higher income individuals were more sensitive to lottery-based (vs. certain) and voucher-type (vs. cash) incentives compared to their lower income counterparts [33]. As this body of research develops, interventionists will be in a better position to match individual characteristics to preferred and/or more effective incentive design features/attributes.

For instance, in the future, older adults may be offered “certain chance” incentives (e.g., 1 in 5 chance of winning $25) given their suspected inclination toward lottery-based interventions [24]; higher income individuals may receive larger incentives (1.2 % or more of disposable income, as has been suggested) [3]; individuals identified as less confident in their ability to exercise may be offered incentives for more achievable, “self-regulatory” behaviors (e.g., wearing a pedometer) as opposed to the attainment of difficult to achieve biometric outcomes (e.g., lose 10 lb) [23]; overweight adults could receive escalating incentives to drive regular exercise over longer periods [34]; and individuals preferring grocery store over iTunes vouchers may receive the credit they prefer and value the most [35]. Collecting relevant sociodemographic and health-related information in future studies will assist with the matching of personal characteristics with more promising incentive approaches.

### Study limitations

The HIP-Q builds on previous attempts to explore incentive acceptability by sampling a wider range of instrumental and affective attitudes and by measuring the effect of program features, including type, source, timing, and certainty of incentive, on the likelihood of incentive program participation. It was not suitable to have all 14 features from Table 1 represented in the HIP-Q, however. Asking prospective participants about all possible incentive design subtleties proved challenging, in part because it was difficult to fully explore feature nuances in a succinct questionnaire and phrase items in a way that was comprehensible to the target group. Rather, the HIP-Q ended up focusing on those features most likely to moderate opinions in a Canadian CR population [21] and increase probability of incentive program participation.

Owing to the complexity of some of the items, the final iterations of the HIP-Q were simplified (using more general categorical judgements) to maintain the psychometric qualities of the questionnaire. Several questions exploring the subtleties of incentive program design were omitted on account of their perceived complexity, leaving several features only superficially explored (#1–3, 6–7, 10, 12–13). Nonetheless, this questionnaire is the first to the authors’ knowledge to examine the impact of multiple incentive program features on acceptability and, in this sense, makes a novel contribution to wellness incentive programming and research/evaluation.

The following limitations should also be noted. Regarding low test-retest reliability during the pilot testing phase of this study, HIP-Q items were completed *following* the completion of several related questionnaires (demographic and health-related surveys), and thus, responder fatigue may partially account for this. For the affective attitude items, the authors suggest that reliability was low because opinions actually shifted over time, rather than CR patients not fully comprehending the questions, since similarly phrased instrumental attitude items demonstrated “Good” reliability. More research examining affective attitudes is needed. The study sample of CR patients was a convenience sample, and thus, the generalizability of the psychometric properties of the HIP-Q is limited. Certainly, as incentives grow in popularity, it will be worth testing the questionnaire among people in different settings (e.g., younger employees in different sectors), especially within the context of workplace wellness programs targeting multiple health behaviors.

Though several steps were taken to ensure that HIP-Q items were psychometrically sound, they are not without limitation. For example, the stem for item #1 includes both “cash” and “healthy vouchers” which may be problematic for two reasons. First, cash and vouchers may not be equally acceptable due to (a) dead weight loss of the voucher if it is for an item the recipient does not value as much as the giver and (b) time discounting associated with future use of the voucher vs. immediate value of cash. Second, the use of the positive word “healthy” before voucher is not balanced by a similarly positive word before cash. Although these issues cannot be fixed post hoc, they should be acknowledged as limitations. Future validation studies will aim to optimize the psychometrics of the HIP-Q and maximize its generalizability.

## CONCLUSIONS

Financial health incentive programs should be carefully designed, considering the range of available features and attributes in the design process, as well as the impact of contextual factors on incentive acceptability and effectiveness. Even subtle variations in incentive program design can have profound effects. The newly developed HIP-Q has the potential to be a useful tool for assessing attitudes of incentives and examining the role of design features in moderating acceptability and uptake. Taken together, the HIP-Q may serve as a practical incentive design tool, used to increase financial health incentive program enrolment and participation. Further research is now needed to examine whether tailoring incentives to preferences assessed by the HIP-Q improves uptake and effectiveness.

## Figures and Tables

**Fig 3:**
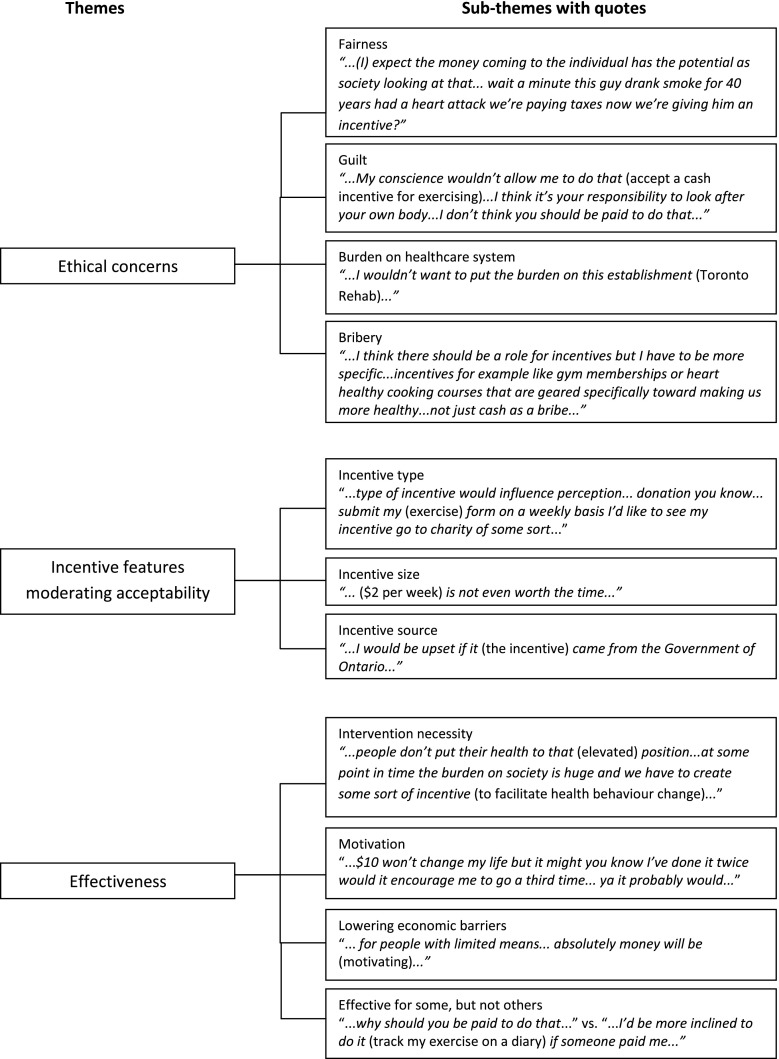
Themes, sub-themes and illustrating quotes from Mitchell et al. [2] focus group study

## Appendix 2. The complete Health Incentive Program Questionnaire

### The Health Incentive Program Questionnaire (HIP-Q)

It may be that a financial health incentive, like getting paid to exercise, could help you start and/or maintain an exercise program. You could be paid in cash, or with healthy vouchers, to do your exercise. You could earn grocery or drug store vouchers, gym discounts, magazine subscriptions or even make charitable donations for exercising regularly! The answers you give in this questionnaire are very important and will help us design a health incentive program *just for you*!
